# Direct Microscopy of Stool Samples for Determining the Prevalence of Soil-Transmitted Helminthic Infections among Primary School Children in Kaduwela MOH Area of Sri Lanka following Floods in 2016

**DOI:** 10.1155/2018/4929805

**Published:** 2018-06-10

**Authors:** Nushka Ubhayawardana, Ishani Gammana Liyanage, H. M. J. C. B. Herath, Uthpala Amarasekera, Tilanka Dissanayake, Sujan de Silva, Nayana Fernando, Sriyani Ekanayake

**Affiliations:** ^1^Department of Parasitology, Faculty of Medicine, SAITM, Malabe, Sri Lanka; ^2^Department of Human Biology and Genetics, Faculty of Medicine, SAITM, Malabe, Sri Lanka; ^3^Department of Forensic Medicine, Faculty of Medicine, SAITM, Malabe, Sri Lanka; ^4^Department of Community Medicine, Faculty of Medicine, SAITM, Malabe, Sri Lanka

## Abstract

A descriptive cross-sectional school based study was carried out to investigate the prevalence of soil-transmitted intestinal helminths and the associated factors among school children in Kaduwela Medical Officer of Health (MOH) area in the Colombo district, which was affected by floods in 2016. The study was conducted in 9 selected schools in Kaduwela MOH area from September 2016 to March 2017. Permission was obtained from the relevant authorities. Grade 1 students were enrolled in the study after obtaining informed written consent from their parents/guardian. Interviewer based questionnaire was administered to gather demographic data and other relevant information. Stool samples were collected and examined by direct wet saline smear. Study population comprised 53.4% male students. None of stool samples were positive for soil-transmitted helminths but 17.4% of students complained of nocturnal itching and parents of 23% of them had seen worms passing out from their children's anus at night. Fourteen stool samples were found to be positive for cyst of* Entamoeba coli*. Majority of parents (69%) stated that their children always wash their hands with soap and water before meals, whereas 26% stated that their children practice this sometimes. Majority of students (88%) washed their hands after going to the toilet. Almost all students (86%) used water sealed toilets and very few used pit latrines (14%). In this study, 67% of students had received anti-helminthic drugs after the floods. These findings suggest that zero prevalence of helminthic infections could be due to anti-helminthic prophylaxis and good health practices. Further studies should be done in this area with a large sample size to investigate the true prevalence of helminthic infections. Students and parents should be educated on* Enterobius vermicularis* infection. The source of water supply should be tested for fecal contamination.

## 1. Introduction

Intestinal nematodes* Ascaris lumbricoides*,* Trichuris trichiura*, and hookworms (*Necator americanus* and* Ancylostoma duodenale*) which infect humans are considered soil-transmitted helminths (STHs). It is included in the World Health Organization list of neglected tropical diseases [1, 2]. The vast majority of infections and burden occurred in Asia, where at least one-quarter (26.4%) of the population was thought to host at least one STH species [3]. STHs can result in a variety of negative health outcomes such as the diarrhea, nutritional deficiencies, and physical and cognitive growth retardation. Most pronounced effects on growth in children can be seen with the heaviest infections but light infections may also contribute to growth deficits if the nutritional status of the community is poor [4]. STHs remain common across the world, especially in the developing nations which primarily consist of low income populations. Low socioeconomic state, poor sanitation, and low educational levels are the main factors influencing the transmission and distribution of STHs [5].

In Sri Lanka,* Ascaris lumbricoides*,* Trichuris trichiura*, and* Necator americanus* infections were recorded recently [6]. The national prevalence of each infection was less than 5%, with a cumulative prevalence of 6.9% for all ascariasis, trichuriasis, and hookworm infections [7]. However, the rates of infections can greatly vary as both host-specific and environmental factors affect the risk of acquiring or harboring heavy intensity helminthic infections [8]. There are higher rates of STHs recorded in coastal areas compared to inland areas in Gampaha District [6]. STH infections are quite common among school children of the plantation sector. Thus, routine deworming is practiced through school health programs. However, according to Gunawardena et al., the prevalence of STH infections among such children can recur after cessation of mass deworming [9].

The distribution, prevalence, and abundance of a parasite are determined by availability of susceptible hosts, as well as environmental thresholds for development [1]. People affected by floods are at greater risk of getting helminthic infections as the flood water can cover the toilet pits and disperse the helminth eggs. Also displaced children have a higher risk of transmitting helminthic infections through concentrated temporary shelters as well as consuming contaminated food and water. Sri Lanka was affected by a severe tropical storm that caused widespread flooding and landslides in May 2016 which was considered a major natural disaster. Colombo was one of the severely affected districts with a potential risk of an outbreak of STHs after the receding of flood water. This study investigates the prevalence of soil-transmitted intestinal helminths and the associated factors among school children in Kaduwela MOH, which was a flood affected area in Colombo district.

## 2. Materials and Methods

### 2.1. Study Population and Study Setting

The study was conducted in 9 selected schools in Kaduwela MOH area (Table 1) from September 2016 to March 2017. All of the selected schools are situated in areas which were affected by the floods.

A total of 373 Grade 1 students were enrolled in the study after obtaining informed written consent from their parents/guardian. Interviewer based questionnaire was administered to gather demographic data and other relevant information. Further, a stool sample was collected from each student. For the sample collection, each parent/guardian was given a labeled stool collecting container and the stool collection procedure was clearly explained. Although 373 questionnaires were filled and stool collecting containers were distributed, only 156 stool samples were returned for examination.

### 2.2. Parasitological Assessment

The stool samples were collected by the investigators on the following day and transported to the Department of Parasitology, Faculty of Medicine, SAITM. The stool samples were preserved in 10% formalin immediately after the collection and stored until examination. Fecal examinations were carried out by observing wet saline smears under the microscope to detect helminth eggs.

Routine parasitological laboratory protocols were followed when examining stool samples. Pinworm infection (*Enterobius vermicularis*) was assessed by the presence of perianal itching and parental observation of passing thread like worms in the night.

### 2.3. Ethical Consideration

The study was conducted after obtaining ethical clearance from Ethics Review Committee of SAITM (Ethical Review no. AS0020 – 16). Further, permission from the Department of Education was also obtained. Finally, prior to conducting the survey, a formal approval was obtained from the principals of each school.

## 3. Results

### 3.1. Demographic Data of Students

Total number of 373 Grade 1 students was included in the study. Study population comprised 53.4% male students (Table 2). Among the student population 55.8% of students belonged to the age group of 5-6 years. Majority of fathers of students were permanent employees (57%), whereas most of the mothers were housewives (71%).

### 3.2. Soil-Transmitted Helminthic Infections

After examining the 156 stool samples by normal saline smears, it was revealed that none of the samples were positive for eggs of soil-transmitted helminths. However, 14 of the stool samples were positive for cysts of* Entamoeba coli*.

### 3.3. Health Practices towards Preventing Parasitic Infections

Among the parents 61% stated that their children always clean their hands with soap and water before meals, while 36% stated that their children practice this sometimes. According to the survey, majority of the students (88%) always clean their hands after using the toilet and almost all students (86%) used water sealed toilets while very few (14%) used pit latrines.

As for the main source of drinking water, 73% of students used tap water. Further, 67% of students were found to consume treated water either by boiling or filtration (Table 3).

### 3.4. Anti-Helminthic Prophylaxis Practiced by the Study Population

Out of the 373 students tested in this study, 67% of students had received anti-helminthic drugs after the floods. The drug was given by public health inspectors at the medical programs conducted in schools. Though majority had received anti-helminthic drugs, 17.4% of students still complained nocturnal itching and parents of 23% of them had seen worms passing out from their anus at night. It was observed that the majority of parents were aware of the importance of anti-helminthic prophylaxis and they have been practicing it on their children regularly.

Among the study population, 170 students were affected by floods and during which 105 of them stayed at their relatives' places while 47 students were at welfare centers. The remaining 18 students stayed at their own residences which were flooded. During this period, all except 13 students used flushed toilets on sharing basis either at the welfare centers or at relatives' places. Further, 27% of affected families consumed self-prepared food while the rest had packed food and bottled water.

### 3.5. Clinical Presentations of Students after the Floods

Among the students affected by floods, 10%, 4%, and 21% had diarrhea, vomiting, and abdominal pain, respectively. Further, 34% of students had skin lesions while 7.6% and 11.7% of students complained of itchiness on face and itchiness in hands or legs, respectively.

## 4. Discussion

The prevalence of STH infections has decreased over the decades according to previous studies conducted in Sri Lanka [6, 7]. Results of this study reported zero prevalence of STH infections in Kaduwela MOH area of Colombo district even after heavy floods in 2016. This suggests the effectiveness of anti-helminthic prophylaxis in controlling intestinal nematodes. However, concentration techniques were not performed for detection of eggs due to financial constraints and this was one of the main limitations in this study.

There are several factors that attribute to transmit helminthic infections [8]. STHs have been found to result in stunting and poor cognitive development. In this study, majority of the parents had school education up to Grade 8 or above and they were aware of parasitic infections (paternal education: 44% passed Grade 8, 26% passed O/L, and 4% passed A/L; maternal education: 42% passed Grade 8, 30% passed O/L, and 3% passed A/L). Similar findings were observed in failure of the parents to respond to a study carried out in Sri Lanka in 2011 [10].

Studies have proven that the prevalence of intestinal helminthiasis was high among people going to open fields for defecation [11, 12]. In this study, 86% of students used water sealed toilets and 14% used pit latrines. Further, majority of students (88%) cleaned their hands always after going to the toilet. More than half of the students were consuming treated water by filtration or boiling. These factors may have contributed towards reducing the prevalence of intestinal helminthic infections.

Though 67% of students received anti-helminthic drugs after the floods, 17.4% of them complained of nocturnal itching and parents of 23% of them had seen worms passing out from their anus at night. We could not confirm the presence of* Enterobius vermicularis* infection due to the failure of the parents to respond and/or reluctance of the parents in doing the tape test early morning on their children. Moreover the research team had difficulties in reaching flood affected households. These were the other limitations we had in this study. But from the symptoms and the observation of worms passing out of the anus we assumed that* Enterobius vermicularis* infection is somewhat common among the study population. Therefore, students and parents should be educated on* Enterobius vermicularis* infection and its transmission as well as prevention.

Previous studies from Sri Lanka have shown the effectiveness of administration of anti-helminthic drugs in reducing transmission of helminths [6]. Findings of this study are in line with those, because even after the flood condition the prevalence of STH was zero. However, skin lesions (34%), itchiness on face (8%), and itchiness in hands or legs (12%) were observed in students who were affected by floods. Further, out of affected students 10%, 4%, and 21% had diarrhea, vomiting, and abdominal pain, respectively, despite the absence of STH infections. These clinical presentations may be caused by bacterial, viral, or fungal etiologies, allergies, dietary changes, indigestion due to consumption of contaminated food obtained from sources other than home, drinking untreated water, exposure to flood water, and living in crowded welfare centers.

Out of the 170 students affected by floods, 47 students stayed at welfare centers with their parents but all were negative for soil-transmitted helminthic infections. This negative prevalence would probably be due to their good health practices in preventing parasitic infections as well as the use of anti-helminthic prophylaxis after the floods. It was observed that 67% of the students had been given prophylaxis either by the school or by parents. It is important to continue the routine deworming which is practiced through school health programs as it is a promising strategy to control the burden of STH infections among school children. Ongoing health education programs along with proper sanitation and personal hygiene are recommended to maintain the zero prevalence of helminthic infections. However, future studies should be done in this area with a large sample size and with standard diagnostic techniques to investigate the actual prevalence of helminthic infections. In this study, students who were suspected of having any health issue including helminthic infections were directed to free clinics of Neville Fernando Teaching Hospital, Sri Lanka, and free treatments were provided. All the parents were advised on how to prevent the STH infections by promoting good health practices. The Department of Education and all of the schools were provided with the results of this survey and the recommendations.

## Figures and Tables

**Table 1:** List of schools selected for the study and number of student enrolled.

School Name	Area	No. of student
Vidyawardena Vidyalaya	Kolonnawa	58

Ediriweera Sarathchandra Vidyalaya	Kolonnawa	19

Kotuwila Gamini Vidyalaya	Kolonnawa	14

Tibet S. Mahinda Vidyalaya	Kolonnawa	20

Ihala Bomiriya Secondary School	Kaduwela	83

Nawagamuwa Sirisumanthissa Secondary School	Kaduwela	45

Kothalawala Primary Model school	Kaduwela	24

Yakala Seelalankara Secondary School	Kaduwela	40

Munidasa Kuarathunga Secondary School	Kaduwela	70

**Table 2:** Demographic data of students enrolled in the study.

Variable		Number	Percentage
Age	4-5 years	6	1.6
	5-6 years	208	55.8
	6-7 years	158	42.4

Gender	Female	173	46.4
	Male	199	53.4

Number of Family members	<3	71	19.0
	4	135	36.2
	5	97	26.0
	>6	69	18.5

Paternal education	Schooling up to grade 5	13	3.5
	Passed grade 8	52	13.9
	Passed O/L*∗*	165	44.2
	Passed A/L*∗∗*	98	26.3
	Higher Education	13	3.5

Paternal occupation	Casual	142	38.1
	Permanent	214	57.4
	Un-employed	16	4.3

Maternal education	Schooling up to grade 5	15	4.0
	Passed grade 8	43	11.5
	Passed O/L*∗*	160	42.9
	Passed A/L*∗∗*	115	30.8
	Higher Education	12	3.2

Maternal occupation	Casual	68	18.2
	Permanent	39	10.5
	Un-employed	265	71.0

*∗* O/L: General Certificate of Education (GCE) ordinary level.

*∗∗*A/L: General Certificate of Education (GCE) advanced level.

**Table 3:** Health practices towards preventing parasitic infections.

Variable	Category	Number	Percentage
Washing hands with soap before meals	Always	227	60.8%
	Sometimes	134	35.9%
	Seldom/ never	12	3.2%

Washing hands with soap after using the toilet	Always	330	88.4%
	Sometimes	39	10.4%
	Seldom/ never	4	1.1%

Washing fruits and vegetables prior to consumption	Always	306	82.1%
	Sometimes	64	17.1%
	Seldom/ never	3	0.8%

Wearing slippers outdoors	Yes	203	54.4%
	No	170	45.6%

Child playing with/ handling soil	Yes	306	82.0%
	No	67	17.9%

Type of toilets used by children	Flush toilet	321	86.1%
	Pit latrine	52	13.9%

Main source of drinking water	Tap water	274	73.4%
	Protected well	76	20.3%
	Unprotected well	12	3.2%
	Public well	2	0.5%
	Tube well	9	2.4%

Drinking water treated by boiling or filtration	Yes	250	67.1%
	No	123	32.9%

## Data Availability

The data used to support the findings of this study are available from the corresponding author upon request.
